# Education Indicators for Internal Medicine Point-of-Care Ultrasound: a Consensus Report from the Canadian Internal Medicine Ultrasound (CIMUS) Group

**DOI:** 10.1007/s11606-019-05124-1

**Published:** 2019-06-25

**Authors:** Anshula Ambasta, Marko Balan, Michael Mayette, Alberto Goffi, Sharon Mulvagh, Brian Buchanan, Steven Montague, Shannon Ruzycki, Irene W. Y. Ma, On behalf of the Canadian Internal Medicine Ultrasound (CIMUS) Group, Anshula Ambasta, Marko Balan, Marcus Blouw, Brian Buchanan, Sharon E. Card, Barry Chan, Janeve Desy, Gabriel Demchuk, Colin R. Gebhardt, Alberto Goffi, Samantha Halman, Brendan Kerr, Irene W. Y. Ma, Leslie Martin, Michael Mayette, Steven J. Montague, Sharon Mulvagh, Jennifer Ringrose, Shannon Ruzycki, Jeffrey P. Schaefer, Jeffrey Yu

**Affiliations:** 1 Division of General Internal Medicine, Department of Medicine, University of Calgary, Calgary, AB Canada; 2 Department of Critical Care, Dalhousie University, Halifax, NS Canada; 3 Internal Medicine and Critical Care Medicine Division, Department of Medicine, Université de Sherbrooke, Sherbrooke, QC Canada; 4 Interdepartmental Division of Critical Care Medicine, Department of Medicine, University of Toronto, Toronto, ON Canada; 5 Division of Cardiology, Department of Medicine, Dalhousie University, Halifax, NS Canada; 6 Department of Cardiovascular Medicine, Mayo Clinic, Rochester, MN USA; 7 Department of Critical Care Medicine, University of Alberta, Edmonton, AB Canada; 8 Division of General Internal Medicine, Department of Medicine, Queen’s University, Kingston, ON Canada

**Keywords:** ultrasound, post-graduate medical education, quality assessment, internal medicine, curriculum/program evaluation

## Abstract

**Background:**

Curriculum development and implementation for internal medicine point-of-care ultrasound (IM POCUS) continues to be a challenge for many residency training programs. Education indicators may provide a useful framework to support curriculum development and implementation efforts across programs in order to achieve a consistent high-quality educational experience.

**Objective:**

This study seeks to establish consensus-based recommendations for education indicators for IM POCUS training programs in Canada.

**Design:**

This consensus study uses a modified nominal group technique for voting in the initial round, followed by two additional rounds of online voting, with consensus defined as agreement by at least 80% of the participants.

**Participants:**

Participants were 22 leaders with POCUS and/or education expertise from 13 Canadian internal medicine residency programs across 7 provinces.

**Main Measures:**

Education indicators considered were those that related to aspects of the POCUS educational system, could be presented by a single statistical measure, were readily understood, could be reliably measured to provide a benchmark for measuring change, and represented a policy issue. We excluded a priori indicators with low feasibility, are impractical, or assess learner reactions. Candidate indicators were drafted by two academic internists with post-graduate training in POCUS and medical education. These indicators were reviewed by two internists with training in quality improvement prior to presentation to the expert participants.

**Key Results:**

Of the 52 candidate education indicators considered, 6 reached consensus in the first round, 12 in the second, and 4 in the third round. Only 5 indicators reached consensus to be excluded; the remaining indicators did not reach consensus.

**Conclusions:**

The Canadian Internal Medicine Ultrasound (CIMUS) group recommends 22 education indicators be used to guide and monitor internal medicine POCUS curriculum development efforts in Canada.

**Electronic supplementary material:**

The online version of this article (10.1007/s11606-019-05124-1) contains supplementary material, which is available to authorized users.

## INTRODUCTION

With increasing evidence and support in using point-of-care ultrasound (POCUS) at the bedside,^1–4^ its application in internal medicine is gaining traction in North America and internationally.^4–7^ In 2018, the American College of Physicians issued an official statement in support of POCUS use for internal medicine.^4^ Similarly, for the practice of hospital medicine, the Society of Hospital Medicine has issued a position statement providing guidance for hospitalists and administrators regarding application, training, assessment, and program management for POCUS.^8^ Internal medicine residency training programs have only recently begun to incorporate POCUS in their curricula. A national survey in 2013 revealed that only 25% of internal medicine residency programs in the USA offered a formal POCUS curriculum.^9^ Since then, a number of programs across the USA have described successful efforts at introducing POCUS to their internal medicine training programs, both in a workshop format^10,11^ and longitudinal curricula.^12–14^

Despite these advances, internal medicine point-of-care ultrasound (IM POCUS) curriculum development and implementation continues to be a challenge globally for many residency training programs. For example, a survey study in Chicago suggests that learners continue to feel incompetent in the use of ultrasound,^15^ and learners in Canada similarly reported low level of IM POCUS skills.^16^ Barriers to IM POCUS education consistently cited in the literature include lack of access to equipment, lack of established curricula, limited availability of educational time, and lack of trained faculty.^9,17–20^ Introducing a novel technology such as POCUS into clinical practice requires significant resources and new infrastructure (e.g., ultrasound machines, image archiving systems), and relies on a limited supply of professionals with expertise. As such, integrating POCUS is expected to be formidable.^21^ Potential solutions to integrate POCUS, therefore, must be engineered to anticipate and overcome these obstacles—a multifaceted approach is necessary.

On a global scale, education is diverse and heterogeneous. To allow for the comparisons of the state of education worldwide, the Organization for Economic Co-operation and Development (OECD) publishes annual results on education indicators.^22^ These indicators characterize education outputs, financial and human resources invested, access to education, and learning environments.^22^ These process, structure, and outcome measures provide timely and quantifiable key information metrics for policy decision-makers and can assist in ensuring quality across programs.^23^

Education indicators are rarely utilized in medical education, given the relative stability in medical education over the past decades.^24^ The introduction of POCUS has, in many ways, produced a technological disruption that is challenging in the current era of stability^25^ and is raising new issues in the geographically diverse landscape of Canada. Establishing standards to define program processes, structure, and outcomes is a crucial step to ensure that POCUS program development is deployed in a thoughtful manner and with broad support. Education indicators provide policy makers and educators such as hospital administrators, program directors, and POCUS faculty with a clear and instructive framework to guide curriculum development, implementation, evaluation, and monitoring efforts. Establishing standardized measures of quality can help advance POCUS education in a number of ways.^26^ First, POCUS educators can improve the design and delivery of their POCUS curriculum by adhering to quality metrics espoused by the education indicators. Second, education indicators can assist policy makers such as hospital administrators and program directors in where to direct necessary resources. Third, by adhering to education indicators, greater uniformity in quality can be achieved across programs. This study seeks to establish consensus-based recommendations for education program indicators for internal medicine POCUS training in Canada.

## METHODS

The Canadian Internal Medicine Ultrasound (CIMUS) group is composed of members who are leaders across Canadian internal medicine residency programs, with POCUS and/or education expertise.^27^ This group previously developed consensus-based recommendations for the components of an internal medicine POCUS curriculum.^27^

In this follow-up project, the CIMUS group held a 4-h consensus meeting concurrent with the proceedings of the Canadian Society of Internal Medicine Annual Meeting in Toronto, ON, on November 4, 2017. The objective was to establish consensus recommendations on IM POCUS education indicators. Members met in person or via teleconference. Two members are representatives from the Royal College of Physicians and Surgeons of Canada’s specialty committees in Internal Medicine and General Internal Medicine, but participated as individuals, rather than as representatives of the specialty committees. The Royal College of Physicians and Surgeons of Canada is the standard setting body for Canadian residency programs. In addition, two members, both general internists with expertise in quality improvement (AA and SR), were invited to provide expert input for quality indicator development processes and frameworks. The meeting was facilitated by one POCUS expert with expertise in medical education and consensus methods (IM), and one internist with expertise in quality improvement (AA).

During the meeting, information regarding education indicators for IM POCUS programs, their definition, purposes, and examples of historical and contemporary uses were presented and discussed.^23,28–31^ Specifically, participants were introduced to the indicator framework of inputs, processes, and outputs.^23,32^ This framework approximates the general standards categories that are currently endorsed by the Royal College of Physicians and Surgeons of Canada, the College of Family Physicians of Canada, and the Collège des Médecins du Québec for evaluation and accreditation of Canadian residency programs.^33^

A refresher of the modified nominal group technique for establishing consensus was provided to the meeting participants.^34^ We planned to conduct no more than three rounds of voting (first round in-person plus no more than two rounds online). All rounds were conducted anonymously and the same participants were invited to vote. Prior to voting, the voting members informally agreed upon characteristics of indicators that were beyond the scope of this study because of poor feasibility, impracticality, or low level of evaluation. Specifically, our group agreed at this time to exclude indicators relating to the quality of specific metrics (e.g., quality of ultrasound machines, quality of teachers, and quality of their teaching) due to low feasibility, indicators relating to patient outcomes due to current impracticality and low feasibility (see Supplementary Online Appendix 1). We also excluded indicators related to learner satisfaction due to low level of evaluation based on the Kirkpatrick four levels of training evaluation.^35^ Specifically, indicators that capture only learner reaction were not considered.

### Candidate Education Indicator Development

The 52 candidate indicators presented at the meeting were drafted 3 months prior to the meeting^32^ by two academic internists (IM, JD), both of whom have completed 1-year dedicated fellowship training in POCUS as well as a post-graduate degree in medical education. These indicators were then reviewed by two members with quality improvement expertise (AA, SR) for additional feedback and to ensure that they met the defining criteria for indicators.^23,31^ Education indicators were defined as those that:

1. Pertain to a significant aspect of the educational system;
2. Can be presented by a single statistical measure;
3. Are readily understood;
4. Can be reliably measured to provide a benchmark for measuring change, and
5. Represent a policy issue.

### Consensus Process

At the meeting, the list of 52 candidate indicators was proposed to the CIMUS group and suggestions on additional indicators were sought from participants. Participants voted on each indicator as to whether it should be included as is, included with modifications, or excluded as an education indicator for IM POCUS programs. During the meeting, all participants voted anonymously online in real time (www.mentimeter.com). Large group discussion occurred with each proposed indicator. However, because of the meeting time limit, our large group size, and the extensive list of indicators to be considered, discussion was not conducted in a round-robin format nor was ranking of indicators sought.^34^ Consensus was defined as agreement by at least 80% of the participants.^36^ Indicators that did not reach consensus at the initial meeting were included in subsequent online voting in Round Two. For indicators that did not reach consensus for “inclusion as is,” but did reach consensus when “include as is” was combined with “include, with modifications,” appropriate modifications were made by two members (IM, AA) to the wording of the indicator statements prior to Round Two, based on comments shared by the participants and supported by supplementary notes taken during the initial meeting.

**Figure 1:**
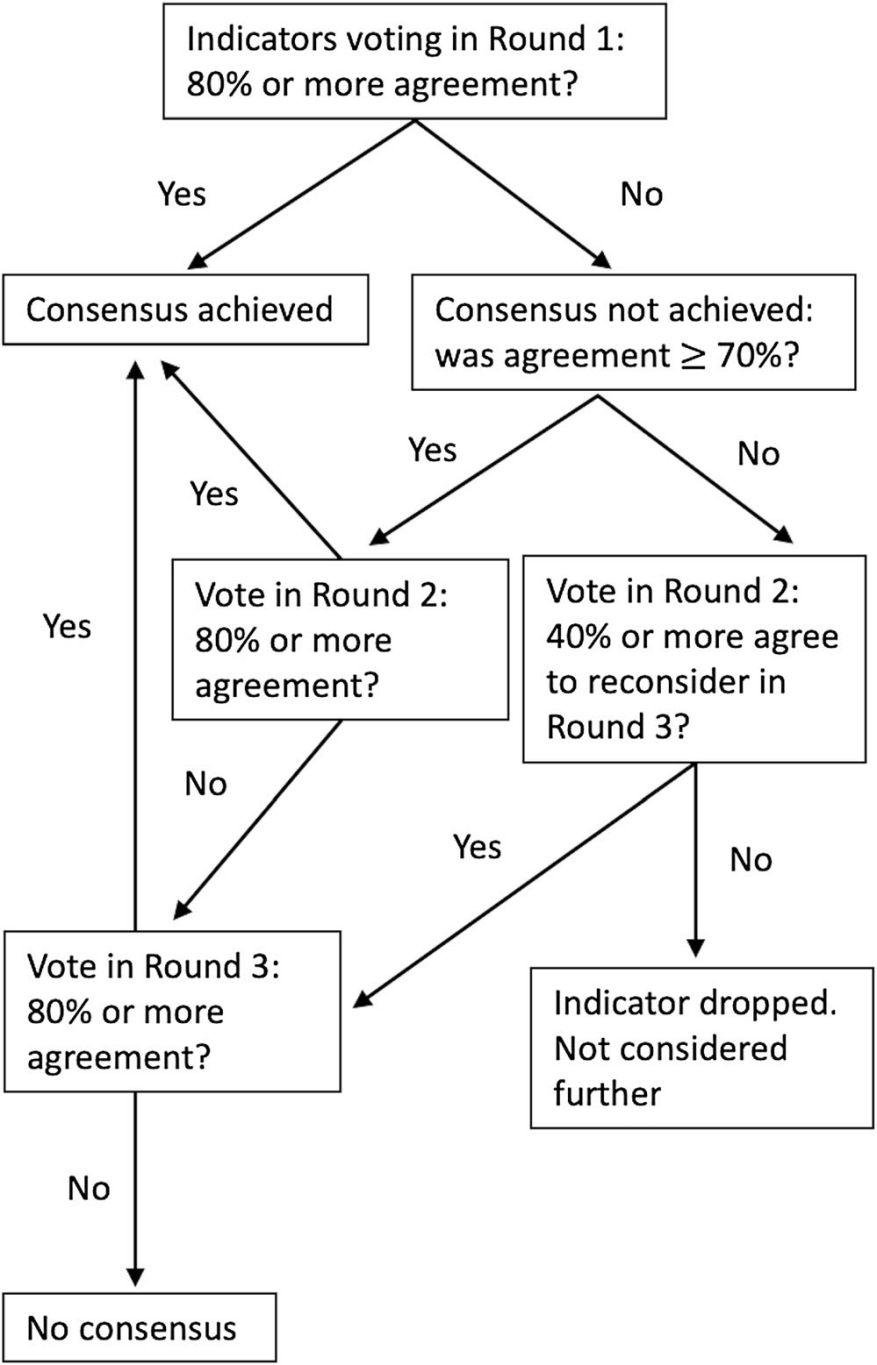
Flow diagram of voting in each round for consensus.

As the majority of items required re-deliberation in Round Two, we categorized results from Round One in the form of ≥ 70% agreement vs. < 70% agreement. We selected this cut-off as it approaches the less conservative median cut-off of 75% used by existing consensus studies.^36^ For indicators with ≥ 70% but < 80% agreement in Round One for inclusion or exclusion, participants were asked to vote in Round Two (Fig. 1). For indicators with < 70% agreement in Round One, participants were asked to flag only those indicators that they wished to reconsider in Round Three. For indicators with ≥ 40% of participants interested in considering further, these were included in Round Three. For indicators with < 40% of participants interested in reconsidering, these were dropped and were not considered further. For Round Three, consensus was considered if 80% or more agreement was reached. Feedback to participants in Round Two was provided in the form of < 70% agreement vs. 70% agreement or more, while exact percentage feedback was given in Round Three. Round Two was conducted approximately 4 months after the in-person meeting, and Round Three occurred 8 weeks after Round Two.

## RESULTS

A total of 22 members participated in the meeting, representing 13 Canadian academic institutions across 7 provinces (Table 1). At the initial meeting, 14 members participated in-person and 8 via teleconferencing.

**Table 1:** Demographics of the 22 Participating Members of the Canadian Internal Medicine Ultrasound Group, Categorized by Academic Institutions, Province, Gender, Subspecialty, Ultrasound, and Teaching Experience

Demographics	*N* (%)
Academic institution	
University of British Columbia	1 (5)
University of Calgary	5 (23)
University of Alberta	2 (9)
University of Saskatchewan	2 (9)
University of Manitoba	1 (5)
Northern Ontario School of Medicine	0
Western University	1 (5)
McMaster University	1 (5)
University of Toronto	1 (5)
Queen’s University	2 (9)
University of Ottawa	2 (9)
McGill University	0
Université de Montréal	0
Université de Sherbrooke	1 (5)
Université Laval	1 (5)
Dalhousie University	2 (9)
Memorial University of Newfoundland	0
Province	
British Columbia	1 (5)
Alberta	7 (32)
Saskatchewan	2 (9)
Manitoba	1 (5)
Ontario	7 (32)
Québec	2 (9)
Nova Scotia	2 (9)
Newfoundland	0
Gender	
Male	14 (64)
Female	8 (36)
Subspecialty	
General internal medicine	16 (72)
Critical care medicine	5 (23)
Cardiology	1 (5)
Year of practice ***using*** ultrasound	
< 1 year	6 (27)
1 to 5 years	7 (32)
6 to 10 years	8 (36)
11 or more	1 (5)
Years of experience ***teaching*** ultrasound	
< 1 year	9 (41)
1 to 5 years	11 (50)
6 to 10 years	1 (5)
11 or more	1 (5)
Years of experience ***assessing*** learner ultrasound skills	
< 1 year	12 (55)
1 to 5 years	9 (41)
6 to 10 years	0
11 or more	1 (5)
Completed 6-month to < 1-year dedicated ultrasound training	2 (9)
Completed a 1-year (or more) dedicated ultrasound fellowship	3 (14)
Completed a fellowship where ultrasound was taught	14 (64)

### Round One

Of the 52 indicators considered, consensus was reached on six to be “included as is” (Supplementary Online Appendix 2), with 46 to be re-considered in Round Two. Of these, 17 indicators reached consensus to be “included with modifications.” None of the indicators reached consensus to be excluded.

### Round Two

All 22 participants voted in this round. A total of 46 indicators were re-considered (Supplementary Online Appendix 2). All 17 indicators from Round One that reached consensus to be “included with modifications” were modified. Of these, 10 indicators were reworded; four indicators on didactic content were proposed to be merged into two (indicators no. 7 with no. 8, no. 9 with no. 10); one indicator on research and program evaluation (no. 49) was proposed to be split into two indicators; and the two indicators on assessments (nos. 50, 52) were proposed to be merged.

Of the 10 indicators which were reworded, all 10 reached consensus to be included. Two additional indicators (nos. 33, 40) that had not previously reached consensus in Round One reached consensus to be included during this round. Five indicators reached consensus to be excluded (nos. 23, 26, 27, 36, 48). For the four indicators on didactic content that were proposed to be merged, consensus was reached for merging two of them (no. 7 and no. 8). Participants did not reach consensus on the merger of the remaining indicators (no. 9 and no. 10; no. 50 and no. 52). Consensus was reached for splitting indicator no. 49.

Of the 18 indicators that had < 70% consensus from Round One to be considered for inclusion, participants were interested in discussing only three of these (nos. 21, 25, 44).

### Round Three

In this final round, where 21 of the members (95%) participated, 14 indicators were considered. Of these, two indicators were to be merged into one (no. 7 and no. 8), and one indicator was to be split into two (no. 49), resulting in a total of 14 indicators considered. Of these, four additional indicators reached consensus to be included (Supplementary Online Appendix 2). Detailed vote results of all three rounds are available in Supplementary Online Appendix 3.

A final list of 22 proposed indicators is presented in Table 2.

**Table 2:** Final 22 Consensus-Based Recommended Education Indicators by the Canadian Internal Medicine Ultrasound (CIMUS) Group, by Category and Initial Indicator Number

Teaching	
5	Total hours of faculty time spent
9	Estimated hours of didactic teaching on image interpretation
10	Estimated hours of didactic teaching on clinical decision/integration
11	Estimated hours of directly supervised hands-on scanning
12	For supervised scans sessions: teacher to learner ratio
14	For independent scanning, feedback mechanisms to learners in place
15	Estimated ratio of trained ultrasound faculty to learners
Learning environment and program organization	
16	Number of dedicated machines accessible to the medical/clinical teaching unit
17	Number of dedicated machines accessible to the medical/clinical teaching unit at each distributed site
19	Has ultrasound program champion(s)
28	Has support from internal medicine residency program director
41	Learner policy in place regarding scope and use of ultrasound
42	Learner scan logs (tracking number of scans)
43	Program has in place suggested target number of scans for each application
Data management and quality assurance	
33	Archiving system in place
34	Quality assurance system in place (images reviewed for quality assurance)
35	Program has minimal criteria in place for acceptable scans
37	Estimated percentage of learner scans reviewed by someone competent to do so
40	Mechanisms in place to deal with incidental findings
Assessment and program evaluation	
49a	Point-of-care ultrasound program evaluation present
50	Assessment processes of image acquisition skills in place
51	Assessment processes of image interpretation in place

## DISCUSSION

Based on the results of our consensus, we recommend that 22 education indicators (Table 2) be considered in the development, implementation, evaluation, and monitoring of IM POCUS training curricula for Canadian internal medicine residency programs. These indicators cover domains including teaching, learning environment and program organization, data management and quality assurance, and assessment and program evaluation. These indicators may serve three overarching purposes: directing curriculum development and implementation efforts, benchmarking curriculum progress over time, and allowing for cross-comparisons and standardization of performances across programs. While only 22 indicators are recommended, it is important to keep in mind that these represent a core number of elements that programs should consider essential to track during implementation and longitudinally; other indicators that did not achieve consensus may also be important to consider.

While our recommended education indicators address a variety of curricular elements, the majority of the recommended indicators relate to learning environment and program organization. Some of these indicators are similar to training and quality assurance processes recommended by policy statements and guidelines from other official bodies.^37–40^ However, our recommendations differ from these in three ways. First, while these other policy statements provide general guidance for educators and training programs, to our knowledge, ours is the first group to recommend actual indicators. These indicators provide quantifiable measures that residency programs can target during POCUS curriculum implementation. Second, existing guidelines from other associations are directed towards specialties where the practice of POCUS has now been fully integrated and its scope well defined.^37,39^ Therefore, explicit guidance and ongoing program monitoring may not be as critical for these fields as their training pathways are already well established. In contrast, IM POCUS is a relatively new field. Explicit guidance from indicators may be more valuable. Lastly, because our indicators were developed by consensus of representatives from the majority of Canadian internal medicine residency programs, the involvement of key stakeholders may help programs across the country produce a more uniform educational landscape.

There are several limitations to our study. First, our group is composed entirely of Canadian educators who are familiar with the current enablers and barriers in the Canadian IM POCUS education system. These indicators may not be generalizable to other settings and may change over time. For example, in settings where educational efforts are directed in a more top-down approach,^5^ the current recommended indicator regarding support from the internal medicine residency program director may be of lesser importance than support from national certifying bodies. Further, Canada only has 17 internal medicine residency training programs. Stipulating uniform use of indicators on a national level for countries with higher number of training programs may be more challenging. Second, from the outset, our group recognized a number of limitations in the scope of the education indicators. The quality of some indicators (e.g., quality of the ultrasound machines, quality of the didactic teaching) may be difficult to capture, given the subjective nature of these measures as well as a lack of available metrics. As such, we acknowledge that our consensus list is not comprehensive. Third is the issue of representation; despite involving national and local internal medicine POCUS leading educators in this study, we do not have provincial ministry and health authority involvement. From an educational perspective, involving only proximal representatives is a limitation. In addition, given that POCUS is a relatively new skill, a number of our experts have limited experience in teaching POCUS. However, the development of education indicators requires expertise in educational principles. To that end, our panel is deliberately diverse to include medical education experts. Fourth, the time frame between the first and second round was rather long. While recommendations on consensus studies do not specify time limits between rounds,^34,36^ the longer this time frame, the longer it would presumably take participants to re-acquaint themselves with the questions,^41^ which may lower intra-rater reliability. Last, operationalization of indicators will need to be better defined. For example, while we intend for indicator no. 5 (total hours of faculty time spent) to encompass total hours of faculty spent on curriculum development as well as delivery, whether or not this indicator can be accurately and feasibly captured must first be established with additional studies. Precise definitions of each indicator will then need to be iteratively established.

### Future Directions

Next steps include operationalization of these indicators and a trial of gathering indicator information on a program-specific level. It is our hope that these indicators can help drive standardization of curriculum development and evaluation efforts in IM POCUS in Canada.

## CONCLUSIONS

The Canadian Internal Medicine Ultrasound (CIMUS) group recommends that these 22 education indicators be used to guide and monitor internal medicine POCUS curriculum development, implementation, evaluation, and monitoring efforts in Canada.

## Electronic Supplementary Material

ESM 1 (DOCX 25 kb)
