# Supplementary material for: Education Indicators for Internal Medicine Point-of-Care Ultrasound: a Consensus Report from the Canadian Internal Medicine Ultrasound (CIMUS) Group
Source: J Gen Intern Med. 2019 Jun 25;34(10):2123–9. doi: 10.1007/s11606-019-05124-1 (PMC6816798; doi:10.1007/s11606-019-05124-1)
Supplement: Supplementary file 1 — (DOCX 25 kb) [file 11606_2019_5124_MOESM1_ESM.docx]

**Supplementary Online Appendix 1: Categories of Indicators Considered, Based on Education at a Glance, 2016 OECD Indicators^1^**

| Indicator Categories | A Priori Decision to Keep vs Drop |
| --- | --- |
| To what level have adults studied? | Keep |
| How many students are expected to complete education? | Keep |
| How many are expected to complete education and what is their profile? | Drop |
| To what extent does parents’ background influence educational attainment | Drop |
| How does educational attainment affect participation in the labour market? | Drop |
| What are the earning advantages from education? | Drop |
| What are the financial incentives to invest in education? | Drop |
| How are social outcomes related to education? | Drop |
| How many students complete education? | Keep |
| How much is spent per student | Keep |
| What proportion of national wealth is spent on education? | Drop |
| How much public and private investment in education is there? | Drop |
| What is the total public spending on education | Keep |
| How much do students pay and what public support do they receive | Drop |
| On what resources and services is education funding spent? | Keep |
| What factors influence the level of expenditure on education? | Drop |
| Who participates in education? | Keep |
| How do early childhood education systems differ around the world? | Drop |
| How many students are expected to enter education? | Drop |
| Who studies abroad and where? | Drop |
| Transition from school to work? | Drop |
| How many adults participate in education and learning? | Drop |
| How much time do students spend in the classroom? | Keep |
| What is the student-teacher ratio and how big are classes? | Keep |
| How much are teachers paid? | Drop |
| How much time do teachers spend teaching? | Keep |
| Who are the teachers? | Keep |
| Who are our school leaders and what do they do? | Keep |

^1^ Organisation for Economic Co-operation and Development. Education at a Glance 2016. OECD Indicators. Available at: http://www.oecd.org/education/skills-beyond-school/education-at-a-glance-2016-indicators.htm

**Supplementary Online Appendix 2. Results of voting from three rounds on education indicators by 22 members. Consensus indicates agreement by at least 80% of the members.**

|  | Indicator | Round 1 | Round 2 | Round 3 |
| --- | --- | --- | --- | --- |
|  | **Output of Education** |  |  |  |
| 1 | Number and % of core learners (Postgraduate years 1-3) with ≥ 4 weeks of ultrasound training | <70% agreement to include | <40% agreement to reconsider | - |
| 2 | Number and % of Fellows (Postgraduate years 4-5) with ≥ 4 weeks of ultrasound training | <70% agreement to include | <40% agreement to reconsider | - |
| 3 | Number and % of learners undergoing advanced training (6 months or more) | <70% agreement to include | <40% agreement to reconsider | - |
|  | **Financial and Human Resources Invested** |  |  |  |
| 4 | Dollars spent on program hardware and software | <70% agreement to include | <40% agreement to reconsider | - |
| 5 | Total hours of faculty time spent | Consensus to include with modifications (91%) | **Consensus to include (95%)** | - |
|  | **Access to Education** |  |  |  |
| 6 | All learners have equal access to learning opportunities | ≥ 70% agreement to include but < 80% consensus | No consensus to include | No consensus to include |
|  | **Learning Environment and Program Organization** |  |  |  |
| 7 | Estimated hours of didactic on physics/safety/knobology | Consensus to include but proposed to be merged with item 8 |  |  |
| 8 | Estimated hours of didactic on scan techniques | Consensus to include but proposed to be merged with item 7 |  |  |
| 7+8 | Round Two: Agree to merge: Estimated hours of didactic on physics/safety/knobology with scan technique? |  | Consensus to merge (95%) | No consensus to include |
| 9 | Estimated hours of didactic on image interpretation | Consensus to include but proposed to be merged with item 10 |  | **Consensus to include (86%)** |
| 10 | Estimated hours of didactic on clinical decision/integration | Consensus to include but proposed to be merged with item 9 |  | **Consensus to include (81%)** |
| 9+10 | Round Two: Agree to merge: Estimated hours of didactic on image interpretation with clinical decision/integrations? |  | < 70% agreement to merge (68%), revote in round 3 as 2 items |  |
| 11 | Estimated hours of directly supervised hands-on scanning | **Consensus to include (86%)** |  |  |
| 12 | For supervised scans sessions: average teacher to learner ratio | Consensus to include with modifications | **Consensus to include (100%)** |  |
| 13 | Estimated hours of protected independent scan time by learners | ≥ 70% agreement to include but < 80% consensus | No consensus to include | No consensus to include |
| 14 | For independent scanning, feedback mechanisms to learners in place | Consensus to include with modifications | **Consensus to include (100%)** |  |
| 15 | Estimated ratio of trained ultrasound faculty to learners | Consensus to include with modifications | **Consensus to include (95%)** |  |
| 16 | Number of dedicated machines accessible to the medical/clinical teaching unit | **Consensus to include (86%)** |  |  |
| 17 | Number of dedicated machines accessible to the medical/clinical teaching unit at each distributed sites | Consensus to include with modifications | **Consensus to include (95%)** |  |
| 18 | No. of shared machines accessible (e.g. intensive care unit / emergency room) to medical/clinical teaching unit learners | <70% agreement to include | <40% agreement to reconsider | - |
| 19 | Has ultrasound program champion(s) | Consensus to include with modifications | **Consensus to include (95%)** |  |
| 20 | Number of ultrasound program champion(s)/lead(s) present | <70% agreement to include | <40% agreement to reconsider | - |
| 21 | At least 1 ultrasound program champion/lead formally trained in ultrasound (6 months or more) | <70% agreement to include | ≥40% agreement to reconsider | No consensus to include |
| 22 | At least 1 ultrasound trained faculty at all distributed sites | <70% agreement to include | <40% agreement to reconsider | - |
| 23 | Program led by subspecialty vs. general internal medicine | > 70% agreement to *exclude* but < 80% consensus | **Consensus to exclude (100%)** |  |
| 24 | If non-internal-medicine led, program has general internal medicine faculty | <70% agreement to include | <40% agreement to reconsider | - |
| 25 | Local training available for pre-existing internal medicine non-ultrasound faculty | <70% agreement to include | ≥40% agreement to reconsider | No consensus to include |
| 26 | Has support from radiology | > 70% agreement to *exclude* but < 80% consensus | **Consensus to exclude (86%)** |  |
| 27 | Has support from cardiology | > 70% agreement to *exclude* but < 80% consensus | **Consensus to exclude (91%)** |  |
| 28 | Has support from internal medicine residency program director | Consensus to include with modifications | Consensus to include (100%) |  |
| 29 | Has support from division chief (of program lead if non-internal medicine lead) | <70% agreement to include | <40% agreement to reconsider | - |
| 30 | Has support from internal medicine division chief | <70% agreement to include | <40% agreement to reconsider | - |
| 31 | Has support from department chair | <70% agreement to include | <40% agreement to reconsider | - |
| 32 | Number of trainee representatives present on the POCUS curriculum committee | > 70% agreement to include but < 80% consensus | No consensus to include | No consensus to include |
|  | **Data Management and Quality Assurance** |  |  |  |
| 33 | Archiving system in place | > 70% agreement to include but < 80% consensus | **Consensus to include (91%)** |  |
| 34 | Quality assurance (QA) system in place (e.g. images reviewed for quality assurance) | **Consensus to include (86%)** |  |  |
| 35 | Program has minimal criteria in place for acceptable scans | **Consensus to include (86%)** |  |  |
| 36 | Estimated hours of quality assurance (QA) image review by faculty | > 70% agreement to include but < 80% consensus | **Consensus to exclude (100%)** |  |
| 37 | Estimated percentage of learner scans reviewed by someone competent to do so | Consensus to include with modifications | **Consensus to include (91%)** |  |
| 38 | Estimated percentage of faculty scans reviewed by another faculty | > 70% agreement to include but < 80% consensus | No consensus to include | No consensus to include |
| 39 | Mechanism in place for machine maintenance | <70% agreement to include | <40% agreement to reconsider | - |
| 40 | Mechanisms in place for incidental findings management | > 70% agreement to include but < 80% consensus | **Consensus to include (91%)** |  |
| 41 | Learner policy in place regarding scope and use of ultrasound | Consensus to include with modifications | **Consensus to include (100%)** |  |
| 42 | Learner scan logs (e.g. numbers being kept track of) | **Consensus to include (86%)** |  |  |
| 43 | Program has in place suggested target number of scans for each application | Consensus to include with modifications | **Consensus to include (100%)** |  |
| 44 | Patient consent policy in place | <70% agreement to include | ≥40% agreement to reconsider | No consensus to include |
| 45 | Simulators available for internal medicine mandated procedures (e.g. central line insertion, thoracentesis) | <70% agreement to include | <40% agreement to reconsider | - |
| 46 | Simulators available for scanning (e.g. FAST, lung etc.) | <70% agreement to include | <40% agreement to reconsider | - |
| 47 | Estimated hours of simulator training for ultrasound-guided procedures | <70% agreement to include | <40% agreement to reconsider | - |
| 48 | Estimated hours of simulator training for scanning | > 70% agreement to *exclude* but < 80% consensus | **Consensus to exclude (86%)** |  |
|  | **Assessment and Program Evaluation** |  |  |  |
| 49 | Point of care ultrasound research/program evaluation present | Consensus to include but proposed to be split into 2 items (49a and 49b) | Consensus to split into 2 items (95%) |  |
| 49a | Point of care ultrasound program evaluation present |  |  | **Consensus to include (100%)** |
| 49b | Point of care ultrasound research present |  |  | No consensus to include |
| 50 | Assessment of image acquisition skills in place | Consensus to include but proposed to merge with item 52 |  | **Consensus to include (90%)** |
| 51 | Assessment of image interpretation in place | **Consensus to include (86%)** |  |  |
| 52 | Assessment of clinical decision making/integration in place | Consensus to include but proposed to merge with item 50 |  | No consensus to include |
| 50+52 | Round Two: Agree to merge: Assessment of image acquisition skills in place with clinical decision making/integration? |  | <70% agreement to merge items |  |

**Supplementary Appendix 3. Results of voting from three rounds on education indicators by 22 members, presented as number (%). Note: not every participant voted on every indicator. Shaded boxes indicate achievement of consensus (agreement by at least 80% of the participants). Black boxes indicate item not considered due to results from preceding round(s).**

|  |  | **Round One** | | | **Round Two** | | | **Round Three** | |
| --- | --- | --- | --- | --- | --- | --- | --- | --- | --- |
|  | Indicator | Include as is | Include with modifications | Exclude | Include | Exclude | < 70% agreement in Round 1 and indicated interest to re-discuss | Include | Exclude |
|  | **Output of Education** |  |  |  |  |  |  |  |  |
| 1 | Number and % of core learners (Postgraduate years 1-3) with ≥ 4 weeks of ultrasound training | 8 (36) | 5 (23) | 9 (41) |  |  | 7 (32) |  |  |
| 2 | Number and % of Fellows (Postgraduate years 4-5) with ≥ 4 weeks of ultrasound training | 11 (50) | 3 (14) | 8 (36) |  |  | 6 (27) |  |  |
| 3 | Number and % of learners undergoing advanced training (6 months or more) | 3 (14) | 4 (18) | 15 (68) |  |  | 4 (18) |  |  |
|  | **Financial and Human Resources Invested** |  |  |  |  |  |  |  |  |
| 4 | Dollars spent on program hardware and software | 7 (32) | 5 (23) | 10 (45) |  |  | 2 (9) |  |  |
| 5 | Total hours of faculty time spent † | 11 (50) | 9 (41) | 2 (9) | 21 (95) | 1 (5) |  |  |  |
|  | **Access to Education** |  |  |  |  |  |  |  |  |
| 6 | All learners have equal access to learning opportunities | 12 (55) | 4 (18) | 6 (27) | 15 (68) | 7 (32) |  | 14 (67) | 7 (33) |
|  | **Learning Environment and Program Organization** |  |  |  |  |  |  |  |  |
| 7 | Estimated hours of didactic on physics/safety/knobology † | 15 (68) | 4 (18) | 3 (14) |  |  |  |  |  |
| 8 | Estimated hours of didactic on scan techniques † | 15 (68) | 4 (18) | 3 (14) |  |  |  |  |  |
| 7+8 | Round Two: Agree to merge: Estimated hours of didactic on physics/safety/knobology with scan technique? § |  |  |  | 21 (95) | 1 (5) |  | 14 (67) | 7 (33) |
| 9 | Estimated hours of didactic on image interpretation † | 15 (71) | 5 (24) | 1(5) |  |  |  | 18 (86) | 3 (14) |
| 10 | Estimated hours of didactic on clinical decision/integration † | 13 (59) | 7 (32) | 2 (9) |  |  |  | 17 (81) | 4 (19) |
| 9+10 | Round Two: Agree to merge: Estimated hours of didactic on image interpretation with clinical decision/integrations? |  |  |  | 15 (68) | 7 (32) |  |  |  |
| 11 | Estimated hours of directly supervised hands-on scanning * | 19 (86) | 1 (5) | 2 (9) |  |  |  |  |  |
| 12 | For supervised scans sessions: average teacher to learner ratio † | 16 (73) | 2 (9) | 4 (18) | 22 (100) | 0 |  |  |  |
| 13 | Estimated hours of protected independent scan time by learners | 12 (55) | 5 (23) | 5 (23) | 15 (68) | 7 (32) |  | 13 (62) | 8 (38) |
| 14 | For independent scanning, feedback mechanisms to learners in place † | 17 (77) | 4 (18) | 1 (5) | 22 (100) | 0 |  |  |  |
| 15 | Estimated ratio of trained ultrasound faculty to learners † | 11 (50) | 7 (32) | 4 (18) | 21 (95) | 1 (5) |  |  |  |
| 16 | Number of dedicated machines accessible to the medical/clinical teaching unit * | 19 (86) | 1 (5) | 2 (9) |  |  |  |  |  |
| 17 | Number of dedicated machines accessible to the medical/clinical teaching unit at each distributed sites † | 14 (64) | 5 (23) | 3 (14) | 21 (95) | 1 (5) |  |  |  |
| 18 | No. of shared machines accessible (e.g. intensive care unit / emergency room) to medical/clinical teaching unit learners | 11 (50) | 4 (18) | 7 (32) |  |  | 6 (27) |  |  |
| 19 | Has ultrasound program champion(s) † | 17 (77) | 2 (9) | 3 (14) | 21 (95) | 1 (5) |  |  |  |
| 20 | Number of ultrasound program champion(s)/lead(s) present | 8 (36) | 3 (14) | 11 (50) |  |  | 4 (18) |  |  |
| 21 | At least 1 ultrasound program champion/lead formally trained in ultrasound (6 months or more) | 7 (32) | 5 (23) | 10 (45) |  |  | 10 (45) | 10 (48) | 11 (52) |
| 22 | At least 1 ultrasound trained faculty at all distributed sites | 5 (23) | 5 (23) | 12 (55) |  |  | 3 (14) |  |  |
| 23 | Program led by subspecialty vs. general internal medicine ‡ | 2 (9) | 3 (14) | 17 (77) | 0 | 22 (100) |  |  |  |
| 24 | If non-internal-medicine led, program has general internal medicine faculty | 7 (32) | 5 (23) | 10 (45) |  |  | 1 (5) |  |  |
| 25 | Local training available for pre-existing internal medicine non-ultrasound faculty | 11 (50) | 4 (18) | 7 (32) |  |  | 9 (41) | 11 (52) | 10 (48) |
| 26 | Has support from radiology ‡ | 2 (9) | 4 (18) | 16 (73) | 3 (14) | 19 (86) |  |  |  |
| 27 | Has support from cardiology ‡ | 2 (9) | 3 (14) | 17 (77) | 2 (9) | 20 (91) |  |  |  |
| 28 | Has support from internal medicine residency program director † | 13 (59) | 5 (23) | 4 (18) | 22 (100) | 0 |  |  |  |
| 29 | Has support from division chief (of program lead if non-internal medicine lead) | 11 (50) | 2 (9) | 9 (41) |  |  | 2 (9) |  |  |
| 30 | Has support from internal medicine division chief | 10 (45) | 5 (23) | 7 (32) |  |  | 4 (18) |  |  |
| 31 | Has support from department chair | 10 (45) | 4 (18) | 8 (36) |  |  | 2 (9) |  |  |
| 32 | Number of trainee representatives present on the POCUS curriculum committee | 11 (50) | 5 (23) | 6 (27) | 15 (68) | 7 (32) |  | 15 (71) | 6 (29) |
|  | **Data Management and Quality Assurance** |  |  |  |  |  |  |  |  |
| 33 | Archiving system in place | 11 (50) | 5 (23) | 6 (27) | 20 (91) | 2 (9) |  |  |  |
| 34 | Quality assurance (QA) system in place (e.g. images reviewed for quality assurance) * | 19 (86) | 2 (9) | 1 (5) |  |  |  |  |  |
| 35 | Program has minimal criteria in place for acceptable scans * | 19 (86) | 3 (14) | 0 |  |  |  |  |  |
| 36 | Estimated hours of quality assurance (QA) image review by faculty ‡ | 8 (36) | 8 (36) | 6 (27) | 0 | 22 (100) |  |  |  |
| 37 | Estimated percentage of learner scans reviewed by someone competent to do so† | 13 (59) | 5 (23) | 4 (18) | 20 (91) | 2 (9) |  |  |  |
| 38 | Estimated percentage of faculty scans reviewed by another faculty | 11 (50) | 5 (23) | 6 (27) | 16 (73) | 6 (27) |  | 16 (76) | 5 (24) |
| 39 | Mechanism in place for machine maintenance | 9 (41) | 3 (14) | 10 (45) |  |  | 7 (32) |  |  |
| 40 | Mechanisms in place for incidental findings management | 12 (54) | 4 (18) | 6 (27) | 20 (91) | 2 (9) |  |  |  |
| 41 | Learner policy in place regarding scope and use of ultrasound † | 14 (64) | 5 (23) | 3 (14) | 22 (100) | 0 |  |  |  |
| 42 | Learner scan logs (e.g. numbers being kept track of) * | 19 (86) | 3 (14) | 0 |  |  |  |  |  |
| 43 | Program has in place suggested target number of scans for each application † | 15 (68) | 6 (27) | 1 (5) | 22 (100) | 0 |  |  |  |
| 44 | Patient consent policy in place | 12 (55) | 3 (14) | 7 (32) |  |  | 9 (41) | 12 (57) | 9 (43) |
| 45 | Simulators available for internal medicine mandated procedures (e.g. central line insertion, thoracentesis) | 5 (23) | 8 (36) | 9 (41) |  |  | 6 (27) |  |  |
| 46 | Simulators available for scanning (e.g. FAST, lung etc.) | 4 (18) | 3 (14) | 15 (68) |  |  | 3 (14) |  |  |
| 47 | Estimated hours of simulator training for ultrasound-guided procedures | 4 (18) | 4 (18) | 14 (64) |  |  | 5 (23) |  |  |
| 48 | Estimated hours of simulator training for scanning ‡ | 3 (14) | 2 (10) | 16 (76) | 3 (14) | 19 (86) |  |  |  |
|  | **Assessment and Program Evaluation** |  |  |  |  |  |  |  |  |
| 49 | Point of care ultrasound research/program evaluation present † | 8 (36) | 10 (45) | 4 (18) |  |  |  |  |  |
| 49a+b | Round Two: Agree to split as: Point of care ultrasound research present and POCUS program evaluation present? § |  |  |  | 21 (95) | 1 (5) |  |  |  |
| 49a | Point of care ultrasound program evaluation present (Split from Round Two for voting in Round Three) |  |  |  |  |  |  | 21 (100) | 0 |
| 49b | Point of care ultrasound research present (Split from Round Two for voting in Round Three) |  |  |  |  |  |  | 7 (33) | 14 (67) |
| 50 | Assessment of image acquisition skills in place † | 14 (67) | 6 (29) | 1 (5) |  |  |  | 19 (90) | 2 (10) |
| 51 | Assessment of image interpretation in place * | 19 (86) | 3 (14) | 0 |  |  |  |  |  |
| 52 | Assessment of clinical decision making/integration in place † | 17 (77) | 3 (14) | 2 (9) |  |  |  | 16 (76) | 5 (24) |
| 50+52 | Round Two: Agree to merge: Assessment of image acquisition skills in place with clinical decision making/integration? |  |  |  | 12 (55) | 10 (45) |  |  |  |

* Consensus achieved to include, as is, in Round One

† Consensus achieved to include, but no consensus to include as is in Round One (for wording changes in Round Two)

‡ Consensus achieved to exclude

§ Consensus achieved to merge/split
